# Supplementary material for: Are carbon emissions trading and green financial instruments synergistic? -Comprehensive quantitative research based on content analysis
Source: PLoS One. 2024 Mar 7;19(3):e0298601. doi: 10.1371/journal.pone.0298601 (PMC10919601; doi:10.1371/journal.pone.0298601)
Supplement: S1 Data — (ZIP) [file pone.0298601.s001.zip › Data/地级市Z_F工作报告.zip!/┴╔─■/╕╖╨┬╩╨/└┤╫╘╣1⁄2╓┌║┼í╛┬φ┐╦╩2╛▌═°í┐_2016.docx]

各位代表：

　　现在，我代表阜新市人民政府向大会报告工作，请予审议，并请列席会议的市政协各位委员和其他同志提出意见。

　　一、“十二五”时期和2015 年工作回顾

　　“十二五”时期，在市委的正确领导下，在市人大、市政协的监督支持下，我们团结带领全市各族人民，全力以赴稳增长促改革调结构惠民生防风险，经济总量持续扩大，收入水平稳步提高，质量效益明显提升，转型振兴迈出坚实步伐。预计地区生产总值达到530 亿元，年均增长6.5%，一般公共预算收入37.2亿元，年均增长4.3%；城镇、农村居民人均可支配收入分别达到22678 元、11100 元，分别年均增长12.3%、11.7%，增速高于经济发展速度。

　　——产业结构进一步优化。工业多元化产业格局基本形成，装备制造、农产品加工、能源三大支柱产业增加值占工业比重75.7%，液压、氟化工、皮革等重点产业集群规模扩大、质量提升、平台完善。服务业增速高于经济发展增速，集聚区初具规模，金融、旅游、电子商务等现代服务业加快发展。200万亩现代农业示范带全面铺开，“畜牧业强市”战略深入实施，新增节水滴灌195万亩。在全国同类资源型城市转型绩效考核中位居第二。

　　——生态建设切实加强。细河生态带初步形成，海州露天矿综合治理全面推进，宜居乡村建设成效初显，污水处理、雨污分流等工程深入实施，主城区化工铸造企业搬迁、地下水源井关闭完成，污染源得到有效治理，流域生态大为改观，空气质量有所提升，城乡环境显著改善。200 万亩经济林工程建成，全市累计植树造林352.7万亩，森林覆盖率32.5% ，城区绿化覆盖率43.1%。草原沙化治理116 万亩。节能减排任务基本完成。

　　——基础设施更加完善。巴新铁路新邱至大板段正式运营，京沈客专阜新段全面建设；新增阜盘、彰通2条高速公路，沈彰开发大道、皮革基地快速通道建成；主城区“六横六纵一环”路网框架基本形成；新建改造城区道路183 条、农村公路2613公里，改造供热管网406公里、供水管网529公里、供气管网125公里。辽西北供水配套工程前期基本完成。城市日供水能力达到35 万吨，污水处理能力29 万吨，城市新增水面41 万平方米。玉龙新城框架基本形成，县城和重点城镇建设稳步推进，常住人口城镇化率达到57%。

　　——改革开放不断深化。市级行政审批事项由882 项减少到258 项，精简率70.7%。清理临时机构和议事机构466个。完成新一轮政府机构改革，事业单位、财税、农村等改革深入推进。工商许可“三证合一”全面实施。全国扶贫改革试验区、国家级高新区获批。与上海有机所等26 家科研院所开展合作。江苏徐工、上海宝钢、中国航天神坤、上海红星美凯龙、大连万达、台湾大润发等知名企业入驻阜新。新增银行机构3家、上市企业2户。累计招商引资到位783 亿元；出口总额9.2亿美元，年均增长17.6%。

　　——人民生活水平稳步提升。完成棚户区改造523.7万平方米、公租房1600 套、廉租房租赁补贴2.1 万户，城市集中连片棚户区、国有工矿棚户区改造基本完成。城镇登记失业率保持4%以内。社保覆盖面扩大、保障能力增强，城乡救助水平持续提高。

　　—— 社会事业实力增强。教育、卫生、文化、体育、养老等公益设施建设力度加大，布局得到优化，功能更加完善，服务质量大幅提高。成功创建东北三省唯一的“全国全民健身示范市”。荣获“中国玛瑙之都”称号。民主法治建设加强，社会管理不断创新，社会和谐稳定。

　　各位代表，过去五年，我国经济社会发展进入新常态，内外部环境都发生了深刻变化，我们准确把握，积极应对，开拓创新。在发展基调上，积极应对经济下行的影响，将稳中求进贯穿工作始终，更加注重提高发展质量效益；在发展方式上，克服传统产业逐渐衰退、新兴产业支撑不足的阵痛，坚持以存量稳增长、以增量调结构，更加注重结构优化、产业升级；在发展措施上，坚持不懈抓招商合作，着力做实项目、做强企业、做大产业，更加注重夯实实体经济基础；在发展导向上，在稳增长的同时，将环境保护、民生改善放在突出位置，更加注重统筹经济效益、社会效益、生态效益和民生效益。这些既是我们“十二五”时期的生动实践，也为今后工作积累了宝贵经验。

　　各位代表，刚刚过去的2015 年，面对严峻复杂的经济形势，我们主动作为，突出经济发展、改革创新、生态建设、民生改善工作主题，经济发展缓中趋稳，社会事业全面进步，民生质量稳步提升。地区生产总值下降9%，城镇、农村居民人均可支配收入分别增长7%、5.1%，城镇登记失业率保持在3.5%以内。

　　（一）稳增长扎实推进

　　把稳增长作为首要任务，制定“四个驱动”实施方案、本地产业产品协作、规范涉企检查、促进商贸流通业发展等政策措施，建立帮扶企业机制，努力提高经济运行质量效益。

　　全力抓好工业经济运行。实施制造业强市行动纲要，促进产业向中高端迈进。重点产业集群基础设施和研发中心、环保设施、市场贸易等配套平台基本建成，骨干企业、拳头产品正在形成。万达铸业、伊利乳业等214 户企业利润实现正增长。规模以上工业增加值130 亿元。氟化工基地获批省级产业技术创新战略联盟，新设立院士工作站2个，新增省级创新平台1个、企业技术中心2个、名牌和商标6个。高新技术企业主营业务总收入增长10%。

　　努力提升服务业水平。实施服务业发展行动计划，连锁经营、专业市场等商业布局基本形成，松涛湖景区晋升省级集聚区。温泉养生、藏传佛教、玛瑙文化等特色旅游影响力不断提升。阜新银行晋升全国城市商业银行百强，盘锦分行开业；在全省率先实施农村土地经营权抵押贷款试点；德尔在深交所创业板上市，天佐羊绒在新三板上市；全年新增贷款77亿元。出台11条政策，促进房地产业平稳发展。成功举办玛瑙、皮革等展会。社会消费品零售总额278亿元，增长7.4%。

　　加快现代农业发展步伐。新建示范带67.6万亩，新开工项目77个。新建、改扩建标准化养殖小区58个。新增节水滴灌27.8 万亩。农田综合机械化水平达到86%。完成高标准农田、农村土地整理46.5万亩。佛寺水库除险加固、柳河治理工程开工。面对严重旱灾，奋力抗灾自救，努力降低损失。

　　（二）重点项目建设取得新进展

　　重大项目支撑作用明显增强。远东铜业、兴隆CBD二期等项目竣工；江苏徐工整车、中国航天神坤液压、上海宝钢集成建筑、江苏中天钛业、辉山乳品加工、振隆提质扩能、上海红星国际广场、紫金财富广场、亚欧建材广场等一批项目加快建设；大连万达广场、封闭母线改造等项目启动。完成固定资产投资210亿元。

　　招商引资取得积极进展。借助“一带一路”、京津冀协同发展战略，以央企沪企民企辽宁行为契机，主动对接国内外500强和行业龙头企业，积极承接产业转移。成功列入国家产业合作与转型升级示范区规划，作为省唯一代表入驻商务部上海基地。市级领导带队赴北京、上海、天津、深圳等地招商40余次，签约五千万元以上项目64个，洽谈对接了一批项目。深化沈阜合作，沈鼓集团风机制造等项目落户。

　　项目工作机制不断完善。建立了领导包保、督查考核、资金奖励三项制度，完善招商项目考核机制，开通重大项目绿色通道。建立项目建设库和储备库，新增亿元以上储备项目115个。

　　（三）城市建设管理水平进一步

　　提高基础设施得到加强。改造供热管网111.4公里、供水管网22公里，新建改造燃气管网134 公里，实施天然气转换5.5 万户。完成东风路等街路改造。友爱桥通车，创业一街跨细河桥主体完工。500千伏输变电工程开工。连接京沈客专彰武段快速铁路前期启动。

　　新一轮城市总体规划修编完成。玉龙新城商业、教育、医疗等布局取得新进展，博乐儿童教育基地等项目启动实施，京沈客专阜新站规划设计完成。

　　城市管理水平提升。城市网格化、属地化管理体制得到巩固，门前“三包”等机制有效落实，环卫工人保障水平提高。创建卫生示范路30条，改造老旧小区39个，综合整治花园市场周边环境，主城区一级清运机械化率达到79%，市容市貌更加清洁、有序。

　　（四）生态环境持续改善

　　“6+1”环境治理工程深入实施。细河综合治理“543”工程全部完工，启动全流域生态综合节点项目，新增湿地15 万平方米。海州露天矿综合治理德国贷款通过国家初审，回填废弃井10座、削坡平盘工程量200 万立方米、植树1.5万株。宜居乡村建设稳步推进，村级规划实现全覆盖，以垃圾整治为重点的村屯环境得到改善。蒙古贞污水二期、温泉城、氟化工基地等污水处理厂建成。煤炭物流园获国家铁路总公司批复。草原沙化治理24万亩，人工造林28.5万亩，新增城市绿地28.8万平方米。主城区空气环境综合整治成效明显，清理整治露天煤场155家。

　　（五）改革开放迈出新步伐

　　调整行政职权420项，推广随机抽查制度，规范事中事后监管。市级非行政许可审批事项全部取消，清理规范30项审批中介服务事项和46件政府文件。建设工程项目审批时限压缩至35个工作日。部门权责清单向社会公布。户籍制度改革稳步推进。出台9条政策支持大众创业、万众创新。新增市场主体1.5万户。

　　积极推进PPP模式，阜盘铁路、供热管网改造等4个项目列为省示范项目。政府债务规范管理。机关事业单位社会养老保险、不动产登记、公车等改革启动。完成公共资源交易662项。

　　新发展农村专业合作社15家。林改确权269万亩。基本药物制度得到巩固，公立医院改革进展顺利。工业用地市场化配置改革试点、农村公路体制改革扎实推进。民营经济贡献增大。

　　对外开放水平提升。赴俄罗斯、台湾等国家和地区开展经贸活动5次，皮革产品出口基地晋升省级，聚宝源服饰、华通管道融入“一带一路”倡议取得突破，全市出口总额2.7亿美元。

　　（六）民生社会事业同步提升

　　省涉及我市的39件实事、市25件实事顺利实施。改造城市棚户区2835户。新增实名制就业4.1万人。企业退休人员基本养老金月人均增加193元。大病保险覆盖面扩大、标准提高。城镇居民医保补助、城乡居民基础养老金、新农合筹资标准分别提高到380元、85元、500元。基本公共卫生服务经费达到人均40元。城市、农村低保标准分别提高到每月460元、每年2832元。实现精准脱贫5.8 万人。投放公积金15 亿元。解决农村3万人饮水安全问题。

　　改造维修校舍40万平方米、薄弱学校12所。市示范性实践学校教学楼、一职专实训楼、实验中学二部综合楼竣工。市全科医生培训基地、社会养老院改扩建工程主体完工。市蒙中教学楼、三院改扩建工程、中心血站和卫生监督所综合楼开工。改扩建乡镇卫生院19家、村卫生室100个，建设无害化厕所5255座。新建改造农村敬老院9 所、互助幸福院50所。

　　圆满完成万人坑死难矿工纪念馆维修保护工程。建成社区书屋85个、乡镇电子阅览室9个。“篮球城”建设和“全民健身年”活动深入开展。

　　（七）政府自身建设加强

　　主动适应新常态，在破解发展难题、转换发展动能、创新工作模式等方面取得突破。深入开展“三严三实”专题教育，全面落实党建和党风廉政建设责任。认真贯彻国家和省市政策措施，建立国发28号文件推进机制，全面落实“四个着力”要求、国务院督查组和省委巡视组反馈意见。深入开展调查研究，广泛听取意见，科学编制“十三五”规划纲要。

　　坚持向省政府和市委报告工作制度；自觉接受人大法律监督和政协民主监督，建议提案办复率100%。强化审计、监察、司法等专门监督，主动接受社会监督、舆论监督。

　　建立重大事项决策咨询、政府法律顾问等制度，出台重大行政决策、重大执法决定合法性审查规则，坚持土地出让价格、国有资产处置等集体审议制度。

　　安全生产形势总体良好。网格化巡查力度加大，重大刑事案件发案率下降，人民群众安全感提升。信访、应急管理得到加强。食品药品安全管理水平稳步提高。社区“两委”换届完成。第三次经济普查获全国先进。民族团结进步事业不断发展，宗教工作和谐稳定。军地合力攻坚，创建全国双拥模范城活动取得显著成效。

　　人防、民防、地方志、档案等工作取得新成绩，外事侨务、对台事务、残疾人事业、红十字会、老龄、慈善等工作迈上新台阶，国税、地税、电力、通信等工作取得新进展，各民主党派、工商联、无党派人士、人民团体等社会各界为经济社会发展作出重要贡献。

　　各位代表、同志们，过去五年，是我市产业发展步伐最坚实的五年，是重大项目引进成果最丰硕的五年，是生态环境改善最明显的五年，是城乡居民得到更多实惠的五年。这是省委、省政府高度重视、全力支持的结果，是市委正确领导、强力推进的结果，是市人大、市政协有力监督、鼎力支持的结果，是全市各族人民团结一心、奋力拼搏的结果。在此，我代表市人民政府，向全市各族人民，向市人大代表、政协委员，向各民主党派、工商联、无党派人士和人民团体，向中省直单位、驻阜部队、武警官兵和政法干警，向关心支持全市发展的老领导们，向所有为阜新发展作出贡献的同志们、朋友们，致以崇高的敬意和衷心的感谢！

　　各位代表，在看到成绩的同时，我们也清醒地认识到，当前经济发展面临的矛盾和问题仍然十分突出。主要是，传统产业支撑能力减弱，新兴产业没有完全成长起来，产业结构深度调整任务艰巨；工业停产半停产企业增多，农业抵御自然灾害能力薄弱，商品房去库存难度较大，投资、消费、出口后劲不足，稳增长压力增大；资源环境约束明显加大，生态改善任务繁重；上级专项资金减少、刚性支出增加、偿债进入高峰期等多种因素叠加，财政收支矛盾十分突出；就业难度增加，脱贫攻坚任务紧迫，保障改善民生压力加大；政府系统一些干部主动适应新常态不够，创新方法、破解难题能力不足。对于这些问题，我们要认真加以解决。

　　二、“十三五”时期基本要求和目标

　　“十三五”时期，是我市全面转型振兴的关键时期，更是全面建成小康社会的决胜阶段。中央政治局审议通过了《关于全面振兴东北地区等老工业基地的若干意见》，明确指出，要切实贯彻“五大发展理念”，深入落实“四个着力”任务，特别是明确资源枯竭地区转型发展到2020年取得显著成效，这是东北老工业基地未来一个时期发展的纲领性文件，更是阜新转型振兴迎来的新一轮重大机遇。我们要认真贯彻党的十八大和十八届三中、四中、五中全会精神，以邓小平理论、“三个代表”重要思想、科学发展观为指导，深入贯彻落实习近平总书记系列重要讲话精神，按照“四个全面”战略布局，牢固树立“五大发展理念”，贯彻“四个着力”要求，以全面转型振兴为主线，以提升质量效益为中心，以做大经济总量、提高人民生活水平为目标，加快推进产业结构优化升级，扎实推进生态宜居城市建设，深入推进改革开放和科技创新，着力保障改善民生，确保如期全面建成小康社会。

　　“十三五”时期，我们要坚持创新发展，把全面创新作为重要支撑，以科技创新为引领，全力推进大众创业、万众创新，培育“六个新增长点”，形成发展新动能；坚持协调发展，把产业结构升级作为主攻方向，以存量稳增长、以增量调结构，同步推进三次产业、“四化”融合、城乡一体化发展，构建现代产业体系；坚持绿色发展，把生态文明作为重要着力点，统筹经济效益、生态效益、社会效益和民生效益，走生产发展、生活富裕、生态良好之路；坚持开放发展，把改革开放作为强大动力，全面深化各项改革，实现“四个驱动”共同发力，构建对外开放新格局；坚持共享发展，把增进人民福祉作为根本，不断提升公共服务供给质量和水平，让发展成果更多惠及人民。

　　今后五年，全市经济社会发展目标是：

　　——经济总量稳步增长。以实现全面小康社会为总目标，经济增速高于全省平均水平，城乡居民收入增速高于经济发展增速，人均地区生产总值、城乡居民收入比2010年翻一番。地区生产总值达到758亿元，年均增长7%，城乡居民收入分别达到32560元、15940元，均年均增长7.5%；一般公共预算收入、固定资产投资等指标同步增长。全面转型振兴取得重大进展。

　　——经济结构明显优化。工业发展进入新阶段，制造业强市加快建设，支柱产业撑起工业经济脊梁，成长性产业向中高端迈进，战略性新兴产业发展壮大，“三四四”产业格局基本形成。现代农业加快发展，质量效益明显提高。现代服务业水平提升，生产性、生活性服务业向专业化、精细化、高品质发展。两化融合、产城互动、三产联动的发展格局加快形成，争创全国产城融合示范市。

　　——生态文明深入推进。空气质量明显好转，流域水质明显改善，矿区生态加快恢复，草原沙化得到遏制，环保设施稳定运行，污染物达标排放，美丽乡村加快建设，城乡环境更加宜居，森林覆盖率达到35%以上，节能减排任务全面完成。

　　——城乡建设协调发展。新型城镇化稳妥推进，空间布局紧凑合理，城市功能更加完善，城市管理服务水平提高，城乡一体化统筹能力增强，智慧城市有序推进，城中村改造基本完成，县城和重点镇承载能力增强，常住人口城镇化率达到61%。

　　——改革开放不断深化。“放、管、服”质量全面提高，市场配置资源的决定性作用有效发挥，对外开放水平显著提升，体制机制灵活顺畅，大众创业、万众创新氛围浓厚，社会活力明显增强，科技贡献率达到60%，民营经济比重达到67%。

　　——人民生活质量提高。深入开展脱贫攻坚，全市9.6万国标贫困人口全部稳定脱贫，贫困县、贫困村全部摘帽。历史遗留问题逐步解决，就业灵活多样，居民收入明显增加，教育、卫生、文化、体育、养老等公共服务供给水平和质量提升，社会保障、救助和福利水平提高。城镇登记失业率控制在5%以内。积极创建国家卫生城。

　　各位代表，经过五年的发展，我们的城市将更有活力，产业多元、功能完善、文明彰显；我们的环境将更加宜居，空气清新、碧水青山、低碳绿色；我们的生活将更有质量，生活宽裕、物质丰富、文化繁荣；我们的社会将更加和谐，保障有力、诚信友爱、快捷高效，我们要朝着全面小康社会美好目标努力奋斗。

　　三、2016年工作安排

　　今年是“十三五”规划开局之年，做好今年工作至关重要。我们要全面贯彻落实市委十一届十次全会暨经济工作会议精神，把握发展形势，保持发展定力，坚定发展信心，努力开创转型振兴新局面。

　　主要预期目标是，地区生产总值增长5.5%，一般公共预算收入增长3%，固定资产投资增长6.5%，出口总额增长5.5%，社会消费品零售总额增长8.2%；城镇、农村居民人均可支配收入分别增长6%、5.5%；城镇登记失业率控制在4.3%以内。

　　（一）以提高质量效益为中心，促进稳增长调结构

　　坚持以企业效益为核心，在存量上优化产品结构，在增量中优化产业结构，在发展中优化经济结构，推动经济平稳运行。

　　深入实施制造业强市行动纲要。推进“三四四”产业发展格局加速形成，加快重点产业集群向中高端迈进。改造提升液压装备、农产品加工、能源产业集群，发展壮大煤化工、氟化工、皮革、板材家居产业集群，着力培育新型建材、钛金属制品、新型电子信息、生物医药等战略性新兴产业。提高重点产业基地的智能化管理水平，完善基础设施、研发检测、企业孵化、金融担保、中介服务、仓储物流等平台。推动江苏徐工整车、中国航天神坤液压、上海宝钢集成建筑等智能制造项目竣工投产。支持现有企业智能化、数字化改造，实施大金重工提质、中国保利民爆器材等项目。围绕大唐煤制天然气项目重组启动，加快配套项目引进建设。抓好中天钛业等重大项目。全力支持阜矿集团转型发展。推动国家级高新区“一区多园”发展，实现规模做大、水平提升。

　　着力抓好工业稳增长、降成本。坚决落实各项政策，切实降低企业生产、运行、流通等环节成本，减轻企业负担，增强企业信心。深化全市产业产品协作，扩大地工产品销售，延伸产业链条，促进本地产业间、企业间融合发展。积极实施帮扶企业等机制，及时解决问题。支持规模以上企业正常生产经营，形成总量增加、企业规模提升的良性局面。抓好停产半停产企业恢复生产工作，盘活现有企业存量资产。支持引导资源类、房地产企业多元化发展实体经济。

　　提升服务业发展水平。加快上海红星国际广场、大连万达广场、兴隆CBD三期、煤炭物流园、紫金财富广场等重大项目建设，豪德农产品、亚欧建材等专业市场建设运营。广发行阜新分行正式营业。大力发展电子商务，完善煤炭、农产品等领域电子交易平台。抓好温泉养生、藏传佛教、玛瑙文化等特色旅游景区开发和产品营销。坚持多措并举，化解房地产库存，促进房地产业健康发展。

　　扩大对外开放。放宽投资领域，优化利用外资结构。实施外贸出口倍增计划，培育农产品、液压、板材等省级出口基地。鼓励企业融入“一带一路”倡议，开拓国际市场。

　　（二）以示范带为载体，加快发展现代农业

　　转变农业发展方式，加强农业新技术、新模式的推广应用，提升农业现代化水平，夯实强农惠农富农基础。

　　全力抓好示范带建设。今年建成78.4 万亩，规范土地经营权流转，推进规模化经营，构建产业、生产、经营体系，培育新型合作组织100个，引进项目100个，全面完成200万亩示范带建设目标，使示范带形成规模、产生效益、辐射带动。

　　优化农业结构。在调减粮食面积、提高单产的基础上，大力发展高效作物，培育壮大一批特色产品专业基地，创建引进一批农产品品牌。争创省级农产品加工园区和现代农业示范区，推进辉山乳品加工、振隆提质扩能、六和肉鸡加工等项目竣工投产。深入实施“畜牧业强市”战略，新建改扩建50个高标准养殖示范场。

　　着力提高农业综合保障能力。新增节水滴灌8 万亩。建设高标准农田24.2 万亩。加快佛寺水库除险加固工程建设。做好人工影响天气工作。扩大农业保险面积。做好第三次全国农业普查。

　　转变农业生产方式。加快农业科研成果应用、转化，发展现代种业，提高机械化水平。发展农村电子商务，新设农产品连锁经营网点10家，完成信息进村入户工程。

　　坚决打好脱贫攻坚战。实施精准扶贫、精准脱贫，扎实推进扶贫改革试验区建设，启动新一轮“集团帮村”，采取产业扶持、社保兜底、教育医疗救助、移民搬迁改造等措施，海州、细河、太平3个城区1284人当年全部精准脱贫，坚决完成全市精准脱贫3.5万人任务。

　　（三）以深化改革为动力，促进大众创业、万众创新

　　落实结构性改革任务，创新体制机制，完善支持大众创业、万众创新政策体系，培育新动能。

　　营造利于“双创”的良好环境。实施创新驱动战略，提升科技创新引领能力，深化与中科院、浙江大学等院校合作，实施科技成果转化项目30项，建设市级工程技术研发中心10个。鼓励支持技术人才、大学生、青年、妇女、新型农民等各类群体创新创业。增强品牌意识，引导企业争创省以上名牌产品和商标。

　　深化行政管理体制改革。继续简政放权，坚持放管结合、优化服务，完善随机抽查机制，加强事中事后监管。动态管理权责清单。进一步清理规范审批中介服务事项。做好工业用地市场化配置改革试点。开展不动产登记工作。

　　深化投融资体制改革。市级将建立产业（创业）投资引导基金，并引导设立创业投资基金、产业投资基金和产业金融投资基金，吸引民间资本。积极建立养老保险风险基金。完善政府偿债准备金制度。推广PPP模式，重点在基础设施、基础产业等领域推广，推进存量项目进行PPP改造。支持企业兼并重组、合资合作，推动阜新银行、田园实业等企业在多层次资本市场上市融资。

　　大力发展民营经济。全面推进“先照后证”、“一证一码”改革。落实各项支持性政策，加快省中小微企业创业基地建设，扶持中小微企业加速成长。

　　深化国企改革。完成各级机关所属企业脱钩改制。引导企业健全公司法人治理结构，推进股权多元化改革。盘活国有存量，积极建立国有资产集团化经营机制。

　　树立过“紧日子”思想，严控“三公”经费，保证正常运转，保证各项改革成本按时支付，保证政府债务不出风险。积极推进公车改革。

　　稳妥推进民生、农村等领域改革。做好土地承包经营权确权登记试点。推广农村土地承包经营权抵押融资。推动县级公立医院药品“零差价”销售。落实中小学教师职称制度改革。

　　（四）以招商引资为抓手，加快项目建设步伐

　　坚持不懈抓招商，始终把项目抓在手上，以招商合作引项目，以项目建设推动经济增长。

　　提高招商实效。主动融入“一带一路”、京津冀协同发展倡议，打造产业转移承接地。以“三四四”工业产业、农业示范带、现代服务业、新型城镇化为重点领域，以国内外500强、行业龙头、上市公司为目标，以央企沪企民企辽宁行、军工企业合作等活动为平台，开展精准招商，深化对接一批项目，着力引进牵动性强、科技含量高、效益好的项目。坚持领导带头招商，建立点对点跟踪机制，实施招商成效考核。发挥企业主体作用，支持引导本地企业开展对外合资合作，加快产品升级换代。

　　继续开展“项目建设年”活动。全年计划新开工投资五千万元以上项目100个，竣工投产100个，储备亿元以上项目100个。完善项目库建设，动态管理市级项目建设库和储备库，围绕“十三五”规划实施，包装、储备一批重大项目，争取更多项目列入省级和国家库。

　　健全项目工作机制。坚持责任分工、定期调度通报等制度，统筹推进各类项目，及时解决困难，提高服务项目水平。转变考核方式，注重单体项目的投资额度、总体进度和综合效益。

　　（五）以环境宜居为目标，加强生态和基础设施建设

　　坚持保护治理并重，实施蓝天碧水青山沃土工程，提升城市功能，建设美丽宜居阜新。

　　抓好生态重点工程。全力推进海州露天矿综合治理，继续实施削坡、平盘、绿化等工程，切实提高治理水平。实施细河治理提升工程，抓好控制性工程、防洪、湿地等项目建设，提高防洪能力、排放水质和生态功能。加快宜居乡村建设，以村容整洁为目的，统筹农村饮水安全、垃圾处理，建成一批示范乡镇、示范村。强化污水处理设施建设运行，确保稳定安全、达标排放。继续推进地方小煤矿关闭。完成人工造林20万亩、水土流失治理14万亩。做好沙化治理，巩固治沙成果。

　　切实改善空气质量。重点实施“黄标车”淘汰、燃煤小锅炉关停、清洁能源推广、绿色交通、工业提标改造等工程，发电企业脱硫脱硝除尘设施稳定运行。做好重点领域节能减排。全面开展环保违规项目清理整顿。

　　抓好基础设施建设。开展阜奈高速、阜盘铁路等项目前期工作，配合京沈客专阜新段及彰武段快速铁路连接线等项目建设。创业一街跨细河桥建成通车，新建青年街桥。开工建设辽西北供水配套工程，中科垃圾发电项目建成运行。积极推进“海绵城市”建设，改造地下管网113.9公里，天然气管网在城市规划区实现全覆盖。整体搬迁改造港华储气罐。改善大修公路192.6公里，完成省下达的村内道路建设任务。

　　推进新型城镇化。严格执行城市总体规划，有序开发各功能区。促进常住人口市民化。进一步完善玉龙新城配套功能，开工建设京沈客专阜新站，并做好周边地区开发和物流、旅游等相关产业规划。温泉城新增开发面积15万平方米，增强集聚能力。重点抓好县城和小城镇建设，推动产城融合发展。

　　加强城市管理和服务。坚持以人为本、源头治理、权责一致原则，进一步完善属地化管理等机制，稳步推进智慧城市建设。提高城市街路规划建设水平，完善停车设施，提升交通通畅能力。加大扬尘治理力度，整顿治理马路市场、占道经营、露天烧烤，搞好城乡结合部、小街小巷等重点区域的环境卫生。以干净安全为目标，做好老旧小区、弃管小区的环境卫生和物业管理。

　　（六）以人民得到实惠为根本，着力保障改善民生

　　坚持共享发展理念，补短板、保基本、兜底线，让人民群众有更多的获得感。

　　积极促进就业。城镇新增就业2 万人。加强对灵活就业、新就业形态的支持，促进劳动者就业。开展职业技能培训，做好就业援助，帮助就业困难者就业。鼓励企业减少裁员。调整全市最低工资标准。

　　完善社会保障和救助体系。依法加强社会保障扩面和征缴，确保各项待遇按时提标、足额发放。推进大病保险全覆盖，整合城乡居民基本医保制度。推进机关事业单位基本养老保险制度改革。完善社会救助体系，落实城乡低保家庭取暖救助全覆盖、残疾人“两项补贴”等制度。推进养老服务业发展，完善医养结合、健康保险等体系。发展慈善事业。

　　做好保障性住房建设，以货币化安置为主，实施棚户区改造777户；公租房100套、租赁补贴500户。改造农村危房3896户。

　　积极构建和谐劳动关系。深入推进平安阜新建设，抓好“七五”普法。完善信访工作机制，依法解决纠纷。落实安全生产责任和制度，提高应急管理能力，严防重特大事故发生。提高食品药品安全管理水平。重视和支持民族地区发展。做好村委会换届。深化双拥工作，巩固和发展军地军民关系。

　　（七）以公共服务均等化为方向，加快社会事业发展

　　加强人才队伍建设。探索建立产学研深度合作的利益分配、人才培养和流动等机制，引进留住优秀人才。支持企业引进创新人才。促进辽宁工大校企合作，通过工学结合等模式，培养一批技能型人才。落实好大学生志愿服务计划。

　　推动教育均衡优质发展。市示范性实践学校教学楼，市蒙中教学楼、一职专实训楼、实验中学二部综合楼等投入使用。阜蒙县、太平区义务教育均衡发展通过省验收。改造维修薄弱学校5所、校舍10万平方米。

　　加强卫生工作。巩固完善新农合制度，参合率保持在99%以上。市三院改扩建工程、中心血站和卫生监督所综合楼投入使用。全面落实“两孩”政策，提高妇幼保健、托幼等服务水平。

　　大力发展公益性文化事业，繁荣文艺精品创作。传承优秀传统文化，弘扬先进文化，凝聚全市力量。市县图书馆、文化馆、博物馆向群众免费开放。巩固“全国全民健身示范市”、“篮球城”建设成果，提高竞技体育水平，促进体育产业发展。

　　（八）以适应新常态为着力点，加强政府自身建设

　　主动适应新常态、顺应新常态，积极作为、履职尽责，推进政府工作再上新台阶。

　　以理念创新引领工作创新。创新体制机制，深刻领会、深入落实“四个全面”战略布局、“四个着力”要求，推进“四个驱动”共同发力，激发动力、释放活力。转换发展新动能，坚持存量增量并重，更加注重质量效益，构建经济转型、产业升级、改革深化、“双创”迸发、“互联网+”等动力多元的支撑体系。创新工作模式，坚持问题导向，破除惯性思维，破解新困难、新问题，适应新常态下科学决策、规范运行、工作创新相融合的新模式。

　　以制度建设推进清正廉洁。注重从制度源头上加大反腐倡廉力度，把纪律挺在法律前面，让权力更加规范高效运行。坚持向省政府和市委报告工作，自觉接受人大法律监督、政协民主监督，认真办理建议提案。注重听取各民主党派、工商联、无党派人士和人民团体的意见建议。按照市委统一要求，做好省委巡视组反馈意见整改工作。推进依法行政，坚持重大资金使用、土地出让价格和国有资产处置集体审议等制度，落实政府法律顾问、重大决策和执法合法性审查、重大事项决策咨询等制度，加快法治政府建设。加强对公共资金、国有资产、国有资源的审计监督。深化政务公开，严格公共资源交易。践行“三严三实”要求，遵守执行《准则》、《条例》，履行“一岗双责”，营造良好政治生态。

　　以转变作风提高行政效能。坚持狠抓落实，认真执行国家、省和市委各项决策部署，守土有责、守土尽责，坚决查处不作为、慢作为等懒政怠政现象。坚持质量效率优先，进一步精简会议文件，优化审批流程，推进网上审批平台建设，缩短审批时限，打造高效快捷的服务环境。坚持底线思维，科学研判工作中可能遇到的各类困难，做充分的思想准备、措施准备，精准发力、重点攻坚。坚持实绩导向，注重工作实效，提高工作质量，建立约谈、追责等工作机制，推动政府系统干部转变作风、奋发有为、干事创业。

　　各位代表、同志们，全面建成小康社会责任重大，使命光荣。让我们在市委的领导下，紧紧团结和依靠全市各族人民，求真务实，奋力拼搏，努力开创阜新转型振兴新局面，为全面建成小康社会而努力奋斗！
